# Decoding the Virtual 2D Map of the Chloroplast Proteomes

**DOI:** 10.1186/s12575-022-00186-8

**Published:** 2022-12-13

**Authors:** Tapan Kumar Mohanta, Yugal Kishore Mohanta, Ahmed Al-Harrasi

**Affiliations:** 1 Natural and Medical Sciences Research Center, University of Nizwa, 616 Nizwa, Oman; 2 Department of Applied Biology, University of Science and Technology Meghalaya, Baridua, Meghalaya 793101 Techno City, India

**Keywords:** Chloroplast, Proteome, Isoelectric point, Molecular weight, 2D

## Abstract

**Background:**

The chloroplast is a semi-autonomous organelle having its own genome and corresponding proteome. Although chloroplast genomes have been reported, no reports exist on their corresponding proteomes. Therefore, a proteome-wide analysis of the chloroplast proteomes of 2893 species was conducted, and a virtual 2D map was constructed.

**Results:**

The resulting virtual 2D map of the chloroplast proteome exhibited a bimodal distribution. The molecular mass of the chloroplast proteome ranged from 0.448 to 616.334 kDa***,*** and the isoelectric point (*pI*) ranged from 2.854 to 12.954. Chloroplast proteomes were dominated by basic *pI* proteins with an average *pI* of 7.852. The molecular weight and isoelectric point of chloroplast proteome were found to show bimodal distribution. Leu was the most abundant and Cys the least abundant amino acid in the chloroplast proteome. Notably, Trp amino acid was absent in the chloroplast protein sequences of *Pilostyles aethiopica*. In addition, Selenocysteine (Sec) and Pyrrolysine (Pyl) amino acids were also found to be lacking in the chloroplast proteomes.

**Conclusion:**

The virtual 2D map and amino acid composition of chloroplast proteome will enable the researchers to understand the biochemistry of chloroplast protein in detail. Further, the amino acid composition of the chloroplast proteome will also allow us to understand the codon usage bias. The codon usage bias and amino acid usage bias of chloroplast will be crucial to understanding their relationship.

**Supplementary Information:**

The online version contains supplementary material available at 10.1186/s12575-022-00186-8.

## Background

The chloroplast is a semi-autonomous organelle in plant cells. It is responsible for photosynthesis and the biosynthesis of several other vital molecules, including amino acids, fatty acids, and terpenoids. The chloroplast was derived from an independent, prokaryotic endosymbiotic ancestor with a small genome. Chloroplast genomes possess three to 273 protein-coding DNA sequences (CDS) [1], and the organelle is fundamental to plant productivity and survival. A large number of chloroplast proteins are associated with photosynthesis and fatty acid biosynthesis. Several chloroplast proteins increase or decrease in abundance as a part of different stress and signaling responses. Therefore, understanding the expression of functional chloroplast proteins is important. Nuclear-encoded proteins are also present in chloroplasts and function in diverse cellular processes. This indicates that the chloroplast proteome is determined by two genomes and is bidirectionally regulated by both the chloroplast and the nucleus. The functional characterization of a protein depends on knowing its sub-cellular localization, co-and post-translational modifications, and enzymatic activity. The field of proteomics focuses on characterizing all the proteins expressed by an organism or tissue. To enable the global identification of proteins, extracted proteins must be first separated by different methods, such as 2D electrophoresis, before their identification by mass spectrometry. Although the genomes of thousands of species have been sequenced, the number of proteins identified and characterized by 2D electrophoresis is very low due to their high level of complexity. Less than 10% of the proteins in the SWISS-PROT database have been identified in 2D gels. This suggests that 2D protein gel electrophoresis cannot be used to provide a comprehensive picture of the proteome. Proteins commonly interact with other proteins, lipids, and nucleic acids. These complex interactions make many proteins challenging to solubilize in an extraction buffer and subsequently separate. Therefore, it is often necessary to separate the protein from its non-protein component so it can be easily separated by isoelectric focusing (IEF) using a wide *pH* gradient. Mass spectrometry analysis of the entire cellular proteome remains a daunting task due to the compartmentalization of proteins in eukaryotic cells and their complex interactions with other molecules. However, the continuing increase in sequenced genomes dramatically increases our ability to identify predicted translated protein sequences and understand protein function. Several different parameters can be used to characterize the complexity of a protein, including its isoelectric point (*pI*), molecular mass, and charge; all of which determine its separation in a 2D gel. In the current study, the complete annotated genomes of more than 2500 species were used to construct a virtual 2D proteome map of the plastome based on their molecular weight and isoelectric point (*pI*). The *pI* and molecular weight of a protein can be sequentially used to separate proteins by 2D electrophoresis. In 2D gel-based electrophoresis, proteins are first separated by using immobilized *pH* gradient (IPG) strips and polyacrylamide gel electrophoresis (PAGE), which is then followed by separation in a second dimension based on molecular mass using SDS (sodium dodecyl-sulfate)-PAGE. These data have been used to construct a virtual 2D proteome map of the chloroplast plastome of plants.

In this study**,** we have delineated the proteomic details of the chloroplast proteome of 2893 species constituting 256,387 protein sequences and constructed a virtual 2D map of the chloroplast proteome. The virtual 2D map of the chloroplast proteome showed bimodal distribution. The average *pI* of the chloroplast proteome was 7.825, and the molecular weight of the chloroplast proteome ranged from 0.448 to 616.334 kDa. Amino acid composition study revealed that Leu was highest and Cys was the lowest abundant amino acid of the chloroplast proteome while Sec and Pyl amino acid was found to be absent.

## Results

### The Molecular Mass of the Chloroplast Protein Ranged from 0.448 to 616.334 kDa

An extensive analysis of the chloroplast proteome, based on the fully-annotated protein sequences of 2893 species, comprising a total of 256,387 protein sequences, revealed that the molecular mass of the chloroplast plastome ranged from 0.448 to 616.334 kDa (Supplementary File 1). The ribosomal protein L16 (accession: AWK02406.1) of *Cercidiphyllum japonicum* (accession: MG605672.1) encoded the smallest protein (0.448 kDa). In comparison, the cell division protein (accession: AID67672.1) of *Nephroselmis astigmatica* (accession: KJ746600.1) was found to be the largest protein (616.334 kDa) present in the chloroplast proteome. Additional low-molecular-mass proteins found in the chloroplast proteome included the ribosomal protein S12 of *Spondias bahiensis* (0.478 kDa, accession: ANI86804.1), acetyl-CoA carboxylase beta subunit of *Carpinus putoensis* (0.713 kDa, accession: APS87155.1), NADH-plastoquinone oxidoreductase subunit 4 of *Trompettia cardenasiana* (0.969 kDa, accession: AMP19627.1), Ycf1 of *Euryale ferox* (1.120 kDa, accession: AUD56613.1), and ribosomal protein L23 of *Lathyrus odoratus* (1.363 kDa, accession: AIL55910.1) (Supplementary File 1). The smallest protein in the chloroplast proteome was comprised of only four amino acids, M-S-L-V (accession: MG605672.1). A few of the other low-molecular-mass proteins with short peptide sequences were M-L-S-E (ribosomal protein S12, accession: ANI86804.1), M-V-F-S-C-K (acetyl-CoA carboxylase beta subunit, accession: APS87155.1), M-C-S-K-I-K-I-F (NADH-plastoquinone oxidoreductase subunit 4, accession: AMP19627.1), M-I-L-K-Y-N-I-L-I (Ycf1, accession: AUD56613.1), and M-I-I-M-L-E-P-G-Y-S-I-P (ribosomal protein L23, accession: AIL55910.1).

**Fig. 1:**
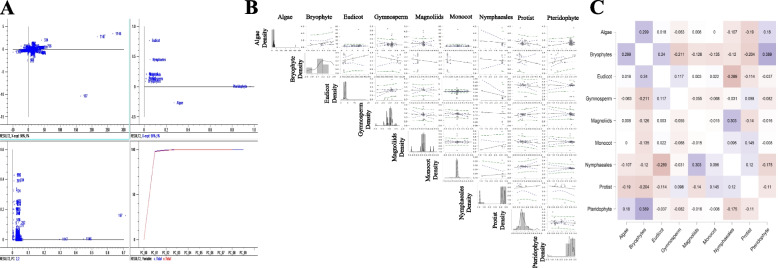
Statistical analysis of low-molecular-mass proteins present in chloroplast proteomes. **A** Principal component analysis (PCoA) of low-molecular-mass proteins in chloroplast proteomes. Low-molecular mass proteins of algae, pteridophytes, nymphaeales, and eudicots are independent, suggesting little to no commonality. **B** Pearson’s correlation analysis of low-molecular-mass proteins in the plant kingdom’s chloroplast proteome of different taxonomic groups. **C** Heat map of Pearson’s correlation values (*p* < 0.05) of low-molecular-mass proteins found in the chloroplast proteome. The majority of low-molecular-mass proteins are negatively correlated with each other

A principal component analysis (PCA) of the low-molecular-mass proteins of the chloroplast proteome revealed that monocots, magnoliids, gymnosperms, and bryophytes share similar low-molecular-mass chloroplast proteins, while the low-molecular-mass proteins of eudicots, nymphaeales, pteridophytes, and algae cluster separately; indicating distinct differences in the low-molecular-mass proteins present within these two groups (Fig. 1). A Pearson correlation analysis (*p* < 0.05) indicated that the low-molecular-mass proteins of eudicots and nymphaeales are negatively correlated (− 0.289), while the low-molecular-mass proteins of bryophytes and algae (0.299), pteridophytes and bryophytes (0.389), bryophytes and eudicots (0.24), and nymphaeales and magnoliids (0.303) were all positively correlated (Fig. 1).

**Fig. 2:**
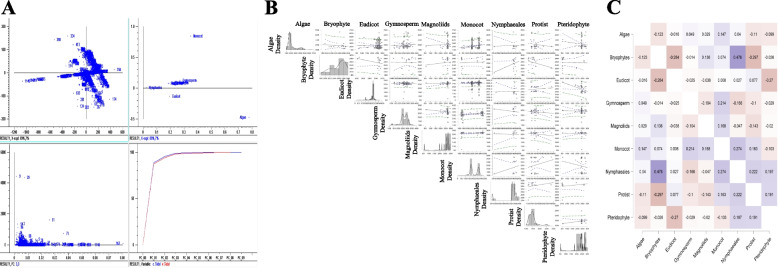
Statistical analysis of high-molecular-mass proteins present in chloroplast proteomes. **A** Principal component analysis (PCoA) of high-molecular-mass proteins in chloroplast proteomes. High-molecular-weight proteins in the chloroplast proteome of different taxonomic groups indicate that monocots, eudicots, and algae are independent, suggesting a lack of commonality in the high-molecular mass chloroplast proteins in these taxonomic groups. **B** Pearson’s correlation analysis (*p* < 0.05) values for high-molecular-mass proteins in the chloroplast proteome of different taxonomic groups. **C** Heat map of the Pearson’s coefficients of high-molecular-mass proteins. A high correlation between nymphaeales and bryophytes is evident, while several others are negatively correlated

The largest identified chloroplast protein (cell division protein) has a molecular mass of 616.334 kDa, and is comprised of 5242 amino acids (Supplementary File 1). Some of the other high-molecular-mass chloroplast proteins were hypothetical chloroplast RF21 (575.771 kDa, accession: AWH11312.1), cell division protein (487.534 kDa, accession: ALO62775.1), hypothetical chloroplast RF1 (485.475 kDa, accession: AHZ11038.1), and Ycf1a (482.348 kDa, accession: GAQ93691.1) (Supplementary File 1). The high-molecular-mass cell division protein was only found in algal species and absent in other species. Principal component analysis of the high-molecular-mass chloroplast proteins revealed that the high-molecular-mass proteins of gymnosperms, bryophytes, magnoliids, protists, and pteridophyte clustered together, while the high-molecular-mass proteins of algae, monocots, nymphaeales, and eudicots clustered independently (Fig. 2). These data suggest commonality in the high-molecular-mass proteins in the lower eukaryotic plant taxa (gymnosperms, bryophytes, magnoliids, protists, and pteridophytes). In comparison, no commonality is present in the higher eukaryotic plant taxa (monocots, nymphaeales, and eudicots). A Pearson’s correlation (*p* < 0.05) analysis revealed that the high-molecular-mass proteins in the bryophytes and nymphaeales were positively correlated (0.476) with each other, while several other groups were negatively correlated (Fig. 2).

**Fig. 3:**
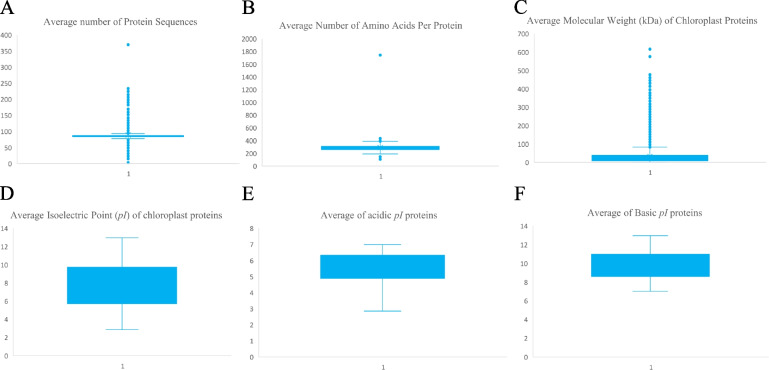
Box and Whisker plot analysis of chloroplast proteomes. **A** An average number of protein sequences. **B** An average number of amino acids per protein. **C** The average molecular mass of chloroplast proteins (kDa). **D** Average isoelectric point, **E** average percentage of acidic *pI* proteins, and **F** average percentage of basic *pI* proteins

Chloroplast proteomes were found to encode a range from 3 to 370 proteins in their proteome. *Pilostyles aethiopica* (eudicot) contained the lowest number of chloroplast-encoded proteins, while *Pinus koraiensis* was found to encode the highest number (370) of chloroplast-encoded proteins. The chloroplast plastome contained an average of 88.749 chloroplast-encoded proteins with an average mass of 32.483 kDa (Fig. 3, Supplementary file 1). Some of the species with a lower number of chloroplast-encoded proteins were *Monoraphidium neglectum* (4), *Pilostyles hamiltonii* (4), *Asarum minus* (7), and *Cytinus hypocistis* (15). Similarly, some of the species encoding a higher number of chloroplast proteins were *Grateloupia taiwanensis* (233), *Grateloupia filicina* (233), *Porphyridium purpureum* (224), *Osmundaria fimbriata* (224), *Lophocladia kuetzingii* (221), and *Kuetzingia canaliculata* (218) (Supplementary file 2). All of the species encoding a high number of chloroplast proteins were algal species (Supplementary file 2). Chloroplast proteomes were found to contain an average of 25,307.87 amino acids per proteome (Supplementary file 2). The highest average protein size was found in *Monoraphidium neglectum,* containing an average of 1743 amino acids per chloroplast protein (Supplementary file 2). The chloroplast proteome of *Grateloupia filicina* encoded the highest number of amino acids with 51,662 (Supplementary file 2). Other species encoding a high number of amino acids in their chloroplast proteome were *Pyropia haitanensis* (50281), *Porphyra purpurea* (50195), *Porphyra pulchra* (50192), and *Palmaria palmata* (50141). The chloroplast proteome of *Pilostyles aethiopica* encodes the lowest number of amino acids with 621 (Supplementary file 2). Other species encoding a low number of amino acids in their chloroplast proteome were *Pilostyles hamiltonii* (911), *Asarum minus* (1727), and *Cytinus hypocistis* (2215) (Supplementary file 2). The average chloroplast protein size was only 288.9613 amino acids (Supplementary file 2). Approximately 33.22% of chloroplast proteins contain ≤100 amino acids, and 15.44% of chloroplast proteins contain ≤50 amino acids. Notably, only 4.69% of chloroplast-encoded proteins contained ≥1000 amino acids.

### The Chloroplast Proteome of *Grateloupia filicina* Is the Heaviest (5854.794 kDa), and Pilostyles Aethiopica Is the Lightest (72.579 kDa)

Approximately 4.8% of chloroplast-encoded proteins had a molecular mass of ≥100 kDa, while 15.502% had a molecular mass ranging from 50 to 100 kDa, and 79.662% had a molecular mass ranging from 0.44 to 50 kDa. The chloroplast proteome of *Grateloupia filicina* was comprised of a total molecular mass of 5854.794 kDa, representing the chloroplast proteome with the greatest molecular mass (Supplementary file 3). Other species containing large molecular mass proteomes included *Grateloupia taiwanensis* (5636.905 kDa), *Pyropia haitanensis* (5636.98 kDa), *Palmaria palmata* (5631.679 kDa), and several other species (Supplementary file 3). The lowest molecular mass chloroplast proteome was found in *Pilostyles aethiopica* (72.579 kDa), followed by *Pilostyles hamiltonii* (106.661 kDa) and *Elytrophorus spicatus* (175.639 kDa) (Supplementary file 3). The average molecular mass of the chloroplast proteome was 2877.533 kDa (Supplementary file 3). The average molecular mass of the chloroplast proteomes of algae, bryophytes, eudicots, gymnosperms, magnoliids, monocots, nymphaeales, protists, and pteridophytes was 3805.064, 2562.121, 2921.544, 2624.771, 2808.423, 2467.242, 2993.64, 2652.881, and 2873.399 kDa, respectively (Supplementary file 3). The average molecular mass of chloroplast proteomes in descending order occurred in the algae (3805.064 kDa) > nymphaeales (2993.64 kDa) > eudicots (2921.544 kDa) > pteridophytes (2873.399) > magnoliids (2808.4232 kDa) > protists (2652.88 kDa) > gymnosperms (2624.77 kDa) > bryophytes (2562.1211) > monocots (2467.241 kDa). Algae contained the species with the greatest molecular mass (3805.064 kDa), while monocots contained the species with the lowest molecular mass chloroplast proteomes (2467.241 kDa) (Supplementary file 3).

### Chloroplast Proteomes Encode a Greater Number of Basic pI Proteins

The *pI* of chloroplast proteins ranged from 2.854 to 12.954 (Table 1, Supplementary file 1). The average e *pI* of all chloroplast proteomes was 7.852 (Fig. 3, Supplementary file 1). The hypothetical plastid protein (accession: CCP38196.1) in *Chondrus crispus* exhibited the lowest *pI* (2.854), while ORF62e (accession: AAO74126.1) in *Pinus koraiensis* had the highest *pI* (12.954) (Supplementary file 1). Other chloroplast-encoded proteins with a low *pI* included the putative ribosomal protein 3 (*pI*: 2.905, accession: AOM65352.1), photosystem I subunit VIII (*pI*: 3.058, accession: AWT39761.1), photosystem I protein I (pI: 3.058, accession: BAK19043.1), cytochrome b6-f complex subunit VI (pI: 3.058, accession: ALM87861.1), and several others (Supplementary file 1). Chloroplast-encoded proteins with a high *pI* were ribosomal protein L34 (*pI*: 12.881, accession: AOM66732.1), ribosomal protein S11 (*pI*: 12.193, accession: API85172.1), ribosomal protein L32 (*pI*: 12.164, accession: ASA34479.1), ribosomal protein S18 (*pI*: 12.12, accession: AHL24798.1), ribosomal protein L36 (*pI*: 12.091, accession: YP_009470691.1), and several others (Supplementary file 1). Among the 256,387 chloroplast proteins analyzed, 56.334% were in the basic *pI* range, 43.611% were found in the acidic *pI* range, and only 0.054% were identified with a neutral (*pI* 7) *pI* (Supplementary file 4). DNA Directed RNA polymerase alpha subunit, a 38.64 kDa protein, was identified as the largest neutral *pI* protein. Although several other proteins with a *pI* 7 were revealed, the Abundance of DNA-directed RNA polymerase alpha subunit was the largest.

**Table 1:** Amino acid composition in the chloroplast proteome of different taxonomic groups of plants and their highest and lowest abundance in the different taxonomic groups

Amino acids	Algae	Bryophyte	Eudicot	Gymnosperm	Magnoliids	Monocots	Nymphaeales	Protist	Pteridophyte	Overall Average	Highest Abundance	Lowest Abundance
Ala	5.850	6.005	5.387	5.781	5.416	5.811	5.431	5.987	6.405	5.591	Pteridophyte	Eudicot
Cys	0.988	1.124	1.139	1.167	1.165	1.141	1.176	0.955	1.199	1.125	Pteridophyte	Protist
Asp	3.982	3.857	4.081	4.336	4.142	3.837	4.185	4.007	4.179	4.067	Gymnosperm	Monocot
Glu	5.307	5.257	5.298	5.688	5.268	5.292	5.349	5.500	5.306	5.312	Gymnosperm	Bryophyte
Phe	5.310	5.594	5.725	5.435	5.510	5.609	5.388	5.503	5.134	5.620	Eudicot	Pteridophyte
Gly	6.206	6.724	6.769	6.623	6.914	7.127	6.8969	6.612	7.092	6.807	Monocot	Algae
His	1.861	2.126	2.354	2.311	2.511	2.362	2.498	1.712	2.256	2.298	Magnoliids	Protist
Ile	8.748	8.363	8.528	8.443	8.569	8.422	8.411	8.654	7.525	8.503	Algae	Pteridophyte
Lys	7.431	6.005	5.547	5.758	5.193	5.394	5.104	7.529	4.962	5.713	Protist	Pteridophyte
Leu	10.64	10.420	10.626	10.387	10.313	10.588	10.281	10.650	10.215	10.590	Protist	Pteridophyte
Met	2.038	2.093	2.339	2.362	2.376	2.360	2.440	2.000	2.067	2.305	Nymphaeales	Protist
Asn	5.836	5.047	4.818	4.560	4.758	4.374	4.658	5.533	4.523	4.800	Algae	Monocot
Pro	3.820	4.284	4.169	4.324	4.207	4.233	4.312	3.727	4.666	4.162	Pteridophyte	Protist
Gln	4.113	3.463	3.559	3.643	3.522	3.474	3.474	3.488	3.200	3.589	Algae	Pteridophyte
Arg	4.800	5.929	6.092	6.138	6.214	6.293	6.267	4.974	6.485	5.988	Pteridophyte	Algae
Ser	7.071	7.652	7.657	7.155	7.871	7.446	7.876	6.994	8.394	7.536	Pteridophyte	Protist
Thr	5.418	5.187	5.069	4.935	5.154	5.249	5.160	5.515	5.281	5.159	Protist	Gymnosperm
Trp	1.266	1.689	1.747	1.732	1.739	1.727	1.751	1.320	1.612	1.683	Nymphaeales	Algae
Tyr	3.652	3.584	3.682	3.554	3.680	3.653	3.724	3.437	3.429	3.659	Nymphaeales	Pteridophyte
Val	5.664	5.588	5.401	5.495	5.539	5.666	5.611	6.166	6.036	5.524	Protist	Eudicot

### Protists Encode more Basic pI Proteins in their Chloroplast Proteomes

**Fig. 4:**
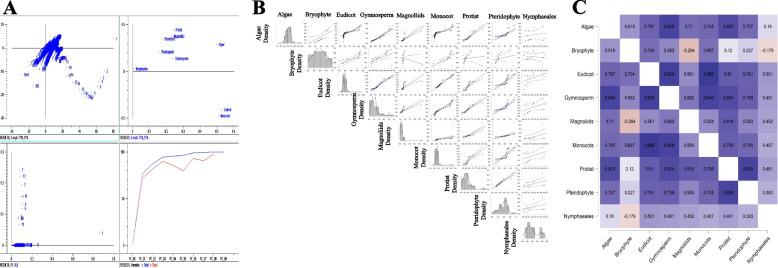
Statistical analysis of basic *pI* proteins in chloroplast proteomes. **A** Principal component analysis (PCoA) of basic *pI* proteins from the chloroplast proteome of different taxonomic groups of plants. The analysis indicated that monocots and eudicots cluster together, suggesting a commonality in these groups’ *pI* of chloroplast proteins. In contrast, algae, protists, and bryophytes are located distantly from the monocot-eudicot cluster. **B** Pearson’s correlation analysis (*p* < 0.05) values of basic *pI* proteins in the chloroplast proteins of different taxonomic groups of plants. **C** Heat map of the correlation between basic *pI* proteins. The analysis indicated several positive correlations between basic *pI* proteins in the chloroplast proteomes of different taxonomic groups of plants

The chloroplast proteomes of protists encoded the greatest percentage of basic *pI* proteins (63.50504%), while the chloroplast proteomes of gymnosperms had the lowest percentage (51.19304%) (Supplementary file 4). The average isoelectric point of the basic *pI* proteins in the overall chloroplast proteome was 9.669 (Fig. 3), while the average isoelectric point of the acidic *pI* proteins was 5.506 (Fig. 3). PCA analysis revealed that the basic *pI* containing chloroplast proteomes of algae and nymphaeales were distant from other groups, while monocots and eudicots clustered together (Fig. 4). The basic *pI* proteins of protists, magnoliids, bryophytes, pteridophytes, and gymnosperms are grouped independently of each other (Fig. 4). Chloroplast proteomes with the highest percentage of basic *pI* proteins, in descending order, were protists (63.505%) > algae (61.936%) > bryophytes (59.380%) > pteridophytes (59.358%) > monocots (55.797%) > eudicots (55.244%) > magnoliids (53.768%) > nymphaeales (52.088%) > gymnosperms (51.193). Correlation analysis indicated that, with the exception magnoliids and bryophytes (− 0.294) and bryophyte and nymphaeales (− 0.179), the basic *pI* proteins of all the other groups were positively correlated (Fig. 4). The algal species, *Prototheca stagnorum,* was found to encode the highest percentage (96.428%) of basic *pI* proteins, followed by *Burmannia oblonga* (95.454%), *Prototheca zopfii* (94.736%), *Burmannia championii* (94.285%), *Neottia listeroides* (94.285%), and *Hydnora visseri* (94.117%) (Supplementary file 4).

**Fig. 5:**
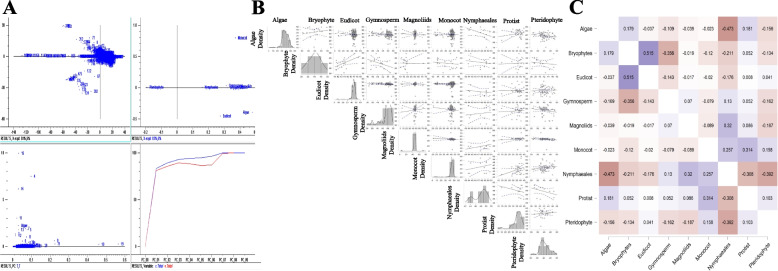
Statistical analysis of acidic *pI* proteins in chloroplast proteomes. **A** Principal component analysis (PCoA) of acidic *pI* proteins from the chloroplast proteomes of different taxonomic groups of plants. The analysis indicated that monocots, eudicots, algae, and pteridophytes locate independently from each other, suggesting a lack of commonality between them. Pearson’s (*p* < 0.05) correlation analysis values of acidic *pI* proteins in the chloroplast proteome of different taxonomic groups of plants. **C** Heat map of the correlation values between other basic *pI* proteins. The analysis indicated that acidic *pI* proteins are negatively correlated with different taxonomic groups of the plants

The chloroplast proteome of *Asarum minus* encoded the lowest percentage (28.571%) of basic *pI* proteins, followed by *Coscinodiscus radiatus* (36.690%), *Schrenkiella parvula* (44.827%), and *Cephalotaxus sinensis* (45.121%) (Supplementary file 4). The chloroplast proteomes of at least 23 species contained more than 90% basic *pI* proteins (Supplementary file 4). Similar to basic *pI* proteins, the chloroplast proteome of gymnosperms had the highest percentage (48.680%) of acidic *pI* proteins. In comparison, the chloroplast proteomes of protists encoded the lowest percentage (36.470%) of acidic *pI* proteins (Supplementary file 4). A principal component analysis indicated that the acidic *pI* proteins of gymnosperms, magnoliids, bryophytes, and protists clustered together, while eudicots, monocots, algae, nymphaeales, and pteridophyte were all located independent of each other (Fig. 5). A Pearson’s correlation analysis of the acidic *pI* proteins in the different taxonomic groups revealed that the acidic *pI* proteins of eudicots and bryophytes (0.515), monocots and protists (0.314), monocots and nymphaeales (0.257), magnoliids and nymphaeales (0.32) were all positively correlated, while the acidic *pI* proteins of algae and nymphaeales (− 0.473), bryophytes and gymnosperms (− 0.356), pteridophytes and nymphaeales (− 0.392), and gymnosperms and pteridophytes (− 0.162) were all negatively correlated (Fig. 5).

The chloroplast proteomes containing the highest percentage of acidic *pI* chloroplast proteins, in descending order, were gymnosperms (48.680%) > nymphaeales (47.911%) > magnoliids (46.145%) > eudicots (44.699%) > monocots (44.219%) > pteridophytes (40.622%) > bryophytes (40.045%) > algae (37.919%) > protists (36.470%). The chloroplast proteome of *Asarum minus* had the highest percentage (71.428%) of acidic *pI* proteins, followed by *Cephalotaxus sinensis* (54.878%), *Pinus tabuliformis* (54.054%), and *Cymbomonas tetramitiformis* (53.94%) (Supplementary file 4). *Prototheca stagnorum* contained the lowest percentage (3.571%) of acidic *pI* proteins, followed by *Burmannia oblonga* (4.545%), *Prototheca zopfii* (5.263%), and *Neottia listeroides* (5.714%).

### The Molecular Weight and pI of the Chloroplast Proteome Exhibits a Bimodal Distribution

**Fig. 6:**
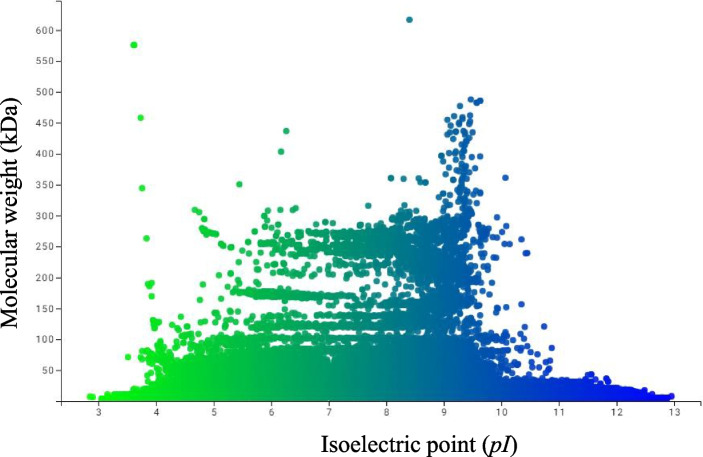
Virtual 2D map of chloroplast proteomes. The X-axis represents the *pI*, and Y-axis represents the molecular mass of different chloroplast proteomes. The overall chloroplast proteome exhibits a bimodal distribution. Basic *pI* proteins are more abundant in chloroplast proteomes than nuclear proteomes; hence the modality shifts towards the basic *pI* range

The isoelectric point and molecular mass values vary greatly among different chloroplast proteomes and may actually exhibit a bimodal distribution (Fig. 6). The calculated mean *pI* of the overall chloroplast proteome was 7.852, and the mean molecular mass was 32.483 kDa. The variance in *pI* was 5.613, which is lower than the mean, while the variance in the molecular mass was 1966.947, which is quite higher than the mean (Supplementary Table 1). The 75th percentile for the calculated *pI* of proteins was 9.736, while the 25th percentile was a calculated *pI* of 5.715 (Supplementary Table 1). The 75th percentile for the calculated molecular mass of chloroplast proteins was 38.95 kDa, while the 25th percentile was calculated to be 9.18 kDa (Supplementary Table 1). The Skewness of the *pI* and molecular mass of chloroplast proteomes was 0.108 and 3.569, respectively, while the kurtosis for *pI* and molecular mass was − 1.246 and 15.282, respectively (Supplementary Table 1). The *pI* exhibited a platykurtic (< 3) distribution, while the molecular mass of chloroplast proteins exhibited a leptokurtic (> 3) distribution. The normal distribution of *pI* for *P*(*X* > 12.954), *P*(*X* < 2.854), *P*(*X* > 7.951), and *P*(*X* < 7.951) was 0.0158, 0.0174, 0.484, and 0.516, respectively (Supplementary Table 1). The normal distribution of molecular mass for *P*(*X* > 616.334), *P*(*X* < 0.448), *P*(*X* > 17.669), and *P*(*X* < 17.669) was 0, 0.235, 0.629, and 0.370, respectively (Supplementary Table 1). These data indicate that the probability of an encoded chloroplast protein with a *pI* above 12.954 is very low (0.0158), and the probability of an encoded protein with a *pI* below 2.854 is less than 0.0174. However, the probability of an encoded protein with a *pI* > 7.951 is very high (0.484). Similarly, the probability of an encoded protein with a molecular mass greater than 616.334 kDa is zero (Supplementary Table 1). Only 126 species (4.35%) of the examined species were found to encode neutral *pI* proteins (Supplementary file 5). *Coeloseira compressa*, *Lobelia anceps*, and *Megaleranthis saniculifolia* encoded two neutral *pI* proteins, while the remaining species were found to contain only one neutral *pI* protein within their chloroplast proteome.

### Chloroplast Proteome Lack Sec and Pyl Amino Acid and the Abundance of Leu Was Highest, and Cys Was Lowest

**Fig. 7:**
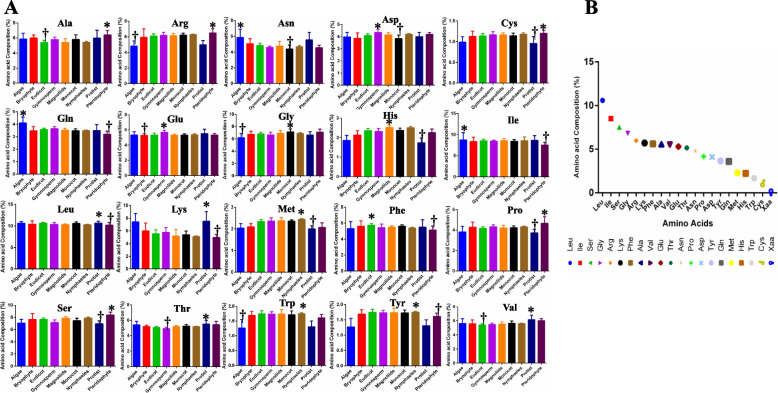
Amino acid composition in chloroplast proteomes. **A** Relative abundance (Percentage) of amino acids in different chloroplast proteomes. Asterisks indicate the highest abundance in the group, and a dagger indicates the lowest abundance. **B** Line graph of the amino acid composition of all 20 essential amino acids and the unknown amino acid Xaa. The graph indicates that Leu is the most abundant and Cys is the lowest abundant amino acid in chloroplast proteomes

**Fig. 8:**
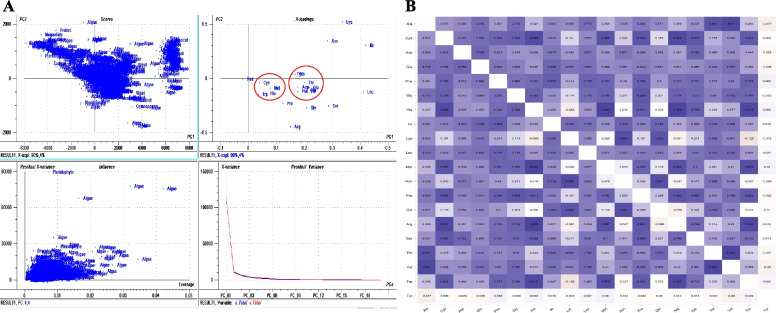
Statistical analysis of amino acid composition in chloroplast proteomes. **A** Principal analysis (PCoA) of amino acid composition in chloroplast proteomes. The analysis indicated that Leu, Ile, Asn, Lys, Pro, Gly, Ser, and Arg amino acids locate independent from each other, while other amino acids cluster in groups; suggesting the differential composition of Leu, Ile, Asn, Lys, Pro, Gly, Ser, and Arg amino acids. **B** Heat map of the Pearson’s correlation analysis values of the amino acid composition in chloroplast proteomes. All of the amino acids, except for Lys and His, were positively correlated

Plastome-wide proteome analysis of amino acid composition revealed that Leu (10.59%) was the most abundant amino acid. At the same time, Cys (1.125) was the least abundant amino acid in the chloroplast proteome (Table 1, Fig. 7, Supplementary file 6). Other high-abundant amino acids in the chloroplast proteome were Ile (8.503%), Ser (7.536%), and Gly (6.807%). Other low abundant amino acids in the chloroplast proteome were Trp (1.683%), His (2.298%), and Met (2.305) (Table 1, Supplementary file 6). The chloroplast proteome was found to encode 50.785% non-polar and 49.197% polar amino acids. Notably, only 0.955% of protist chloroplast proteins contain Cys, and only 0.988% of algal chloroplast proteins contain Cys. The percentage of algal chloroplast proteins containing Arg was 4.8 and 4.97% in protists, which was considerably lower relative to other taxonomic groups (Table, Fig. 7). The highest and lowest abundance of various amino acids in different taxonomic groups are indicated by an asterisk (*) and a dagger (†), respectively, in Fig. 7. None of the analyzed chloroplast protein sequences were found to contain Sec selenocysteine (Sec), and a few encoded Xaa (unknown), B (Asx, codes for Asn or Asp), and J (Xle, codes for Leu or Ile) (Supplementary file 1). At least 108 species contained Xaa, six contained Asx, and eight contained Xle amino acids. The amino acid pyrrolysine, and selenocysteine, were also not found in the chloroplast proteome. The highest and lowest abundant amino acids in many individual species were also determined (Table 2). Most of the species listed in Table 2 were algae or protists and exhibited significant variation in amino acid composition. For example, although the average Percentage of Leu in the chloroplast proteome was 10.590% (Table 1), the Percentage of Leu was 12.385% in the chloroplast proteome of *Codonopsis lanceolata* (Table 2). Similarly, the Percentage of Ile in the chloroplast proteome was 8.503% (Table 1), while the percentage of Ile in *Choreocolax polysiphoniae* was 14.555% (Table 2). The chloroplast proteome of *Pilostyles aethiopica* does not contain Trp and may have lost the genes responsible for encoding this amino acid. A PCA analysis revealed that Leu, Ile, Lys, Asn, and Ser are independent of each other, while Cys, Met, His, and Trp cluster together (Fig. 8). Similarly, Tyr, Gln, Thr, Glu, Asp, Phe, Val, and Gly also cluster together, reflecting their similar percentage of abundance in the proteome. A Pearson’s correlation analysis (*p* < 0.05) of amino acid composition was conducted to better understand their abundance in the chloroplast proteome. Results indicated that a maximum of the chloroplast encoded amino acids were positively correlated with each other, with a few exceptions (Fig. 8). The abundances of Cys, Met, His, Tyr, Gln, Thr, Glu, Asp, Phe, Val, Gly, and Trp were found to be correlated (Fig. 8). A few amino acid combinations exhibited a negative correlation, including Lys and His (− 0.083), Lys and Trp (− 0.128), Lys and Arg (− 0.061), Asn and Tyr (− 0.004), Asn and Trp (− 0.027), Arg and Asn (− 0.047), Gln and Arg (− 0.066), Tyr and Lys (− 0.015), Pro and Tyr (− 0.022), and Tyr and Val (− 0.06) (Fig. 8).

**Table 2:** Highest and lowest percent abundance of amino acids in the chloroplast proteomes of different plant species

Amino Acids	Highest percentage (%)	Name of the species with highest abundance	Lowest percentage (%)	Name of the species with lowest abundance	Variance
Ala	8.678	*Trebouxiophyceae sp*	2.038	*Hydnora visseri*	0.299
Cys	1.786	*Sciaphila densiflora*	0.693	*Monomastix sp*	0.0076
Asp	4.927	*Monotropa uniflora*	2.528	*Cytinus hypocistis*	0.07
Glu	8.304	*Monotropa uniflora*	3.882	*Cytinus hypocistis*	0.079
Phe	5.61	*Abeliophyllum distichum*	4.666	*Zygnema circumcarinatum*	0.161
Gly	9.52	*Selaginella kraussiana*	3.039	*Hydnora visseri*	0.255
His	3.244	*Selaginella moellendorffii*	0.768	*Pilostyles hamiltonii*	0.048
Ile	14.555	*Choreocolax polysiphoniae*	2.438	*Carapa guianensis*	0.497
Lys	13.846	*Hydnora visseri*	2.879	*Selaginella kraussiana*	0.967
Leu	12.385	*Codonopsis lanceolata*	6.895	*Selaginella moellendorffii*	0.107
Met	3.03	*Pinus koraiensis*	1.3	*Hydnora visseri*	0.024
Asn	9.734	*Hydnora visseri*	2.94	*Codonopsis lanceolata*	0.419
Pro	8.162	*Selaginella moellendorffii*	1.932	*Pilostyles aethiopica*	0.087
Gln	5.714	*Rhipilia penicilloides*	1.207	*Pilostyles hamiltonii*	0.078
Arg	8.868	*Allotropa virgata*	3.545	*Ulva flexuosa*	0.309
Ser	9.366	*Monoraphidium neglectum*	5.296	*Monotropa uniflora*	0.195
Thr	6.748	*Hafniomonas laevis*	2.524	*Pilostyles hamiltonii*	0.061
Val	7.479	*Alveolata* sp.	3.321	*Hydnora visseri*	0.124
Trp	2.453	*Chromera velia*	0	*Pilostyles aethiopica*	0.04
Tyr	6.27	*Prototheca zopfii*	2.44	*Trebouxiophyceae sp*	0.048

## Discussion

Plant cells and protists contain a semi-autonomous chloroplast organelle that encodes a small proteome, consisting of a dynamic range of proteins that vary in molecular mass and isoelectric point. The largest protein (616.334 kDa) identified in the chloroplast proteome was a cell division protein and is quite smaller than the largest nuclear-encoded protein in plant cells. Presently, the largest protein encoded in plant cells is a putative polyketide synthase type-I protein with a molecular mass of 2236.8 kDa [2]. Chloroplast proteomes were found to encode a range from 3 to 370 proteins, while the nuclear genome encodes from 6033 (*Helicosporidium* sp.) to 248,180 (*Hordeum vulgare*) protein sequences [2]. The largest chloroplast-encoded proteome in the plant kingdom is 9,857,470.162 kDa (*Hordeum vulgare*), which is 1683.657 times larger than the chloroplast proteome of 5854.794 kDa in *Grateloupia filicina*. The average molecular mass of nuclear-encoded proteomes in the plant kingdom is 1,918,027.187 kDa, which is 666.552 times larger than the average molecular mass of the chloroplast proteome (2877.533 kDa). Chloroplast proteomes encode an average of 88.749 proteins per chloroplast (Fig. 3), while the nucleus encodes an average of 40,469.47 proteins, which is 455.999 times greater than the chloroplast proteome. In algae, the chloroplast proteome encodes larger proteins relative to other taxonomic groups and also has a higher number of proteins. It is reported that chloroplasts originated approximately 1.2 billion years ago as cyanobacterial endosymbionts within a eukaryotic host cell [3]. Later, the endosymbiont genome underwent an enormous reduction in its genome size, decreasing the number of encoded proteins to a range of 3–370 [1].

In contrast, the cyanobacterial genome encodes several thousand proteins [4]. Although it is commonly assumed that the chloroplast maintained its genetic autonomy, this does not seem to be the case. Chloroplasts have frequently lost genes and genetic content and transferred genes to the nucleus [1]. During evolution, genes have been transferred from an ancestral chloroplast to the nucleus and are translated into the cytosol, where they are properly expressed and targeted for import into the chloroplast with the aid of a transit peptide. Our studies have established that almost all chloroplast protein-encoding genes can be found as a nuclear genes in one or more species [1]. Approximately 18% of the nuclear genes in *Arabidopsis thaliana* have been reported to be inherited from cyanobacteria [5]. This observation is explained by the common phenomenon of an exchange of genetic material between the endosymbiont chloroplast and the nucleus. However, the question arises: why protein-encoding genes from the chloroplast have been transferred and merged with the nuclear genome? Is the genomic organization of the chloroplast genome unsuitable for the proper expression and processing of chloroplast-encoding genes inside the eukaryotic cell? The nucleus regulates the chloroplast, so concomitant to this regulation, it may have been more efficient for the chloroplast genes to be transferred to and expressed by the nucleus.

The chloroplast proteome encodes small peptides, with the smallest identified peptide being comprised of M-S-L-V amino acids. This tetrapeptide has a molecular mass of 0.448 kDa, and in comparison, the smallest nuclear-encoded peptide is also a tetrapeptide (M-I-M-F) with a molecular mass of 0.54 kDa [2]. The low molecular mass tetrapeptide identified in the chloroplast proteome of *Cercidiphyllum japonicum* was not found in other species, and the cellular and molecular function of this tetrapeptide are unknown. One of the small molecular mass peptides identified in the nuclear-encoded proteome of plant cells is the cytochrome b6/f complex subunit VIII [2], which is also encoded in the chloroplast proteome (Supplementary file 5). Glutathione is the smallest reported peptide composed of three amino acids (tripeptide) G-S-H [6]. Although nuclear-encoded small peptides in the plant kingdom contain glutathione, chloroplast-encoded small peptides contain Ser (S), an amino acid similar to glutathione. Polypeptides with fewer than 100 amino acids are categorized as small peptides, and 33.22% of the proteins encoded by the chloroplast proteome are composed of ≤100 amino acids. The small peptides play a role in cell signaling, cell growth, and DNA damage response [7–10]. Tri, tetra, and pentapeptides are involved in diverse signaling processes [11, 12]. The tetrapeptide G-E-K-G is associated with the formation of the extracellular matrix [13], the pentapeptide E-R-G-M-T induces the expression of the *srfA-lacZ* gene in *Bacillus subtilis* [14], and A-R-N-Q-T plays a role in sporulation [14]. A previous study reported that the average size of plant proteins is smaller than animal proteins [2]. In the plant kingdom, the average length of nuclear-encoded proteins is 424.34 amino acids, while the average size of chloroplast-encoded proteins is 288.9613 amino acids. The average length of eukaryotic proteins has been reported to be 472 amino acids [15], which is 183.038 amino acids greater than the average length of chloroplast-encoded proteins. Although the average size of chloroplast-encoded proteins is very low relative to nuclear-encoded plant and animal proteins, the chloroplast genome of *Monoraphidium neglectum* encodes an average of 1743 amino acids per protein and was found to only encode a total of four protein sequences.

The chloroplast proteome was found to contain a higher percentage of basic *pI* proteins (56.334%) relative to the nuclear-encoded proteins, the latter of which has been reported to encode a higher percentage (56.44%) of acidic *pI* proteins. The average *pI* of nuclear-encoded acidic proteins is 5.62 [2], slightly higher than the average *pI* of acidic chloroplast proteins (5.506). The average *pI* of basic proteins in the chloroplast proteome is 9.669, slightly higher than the average *pI* (8.37) of basic, nuclear-encoded proteins in the plant kingdom. The *pH* of chloroplasts ranges from 7.8 to 8.2 [16], and the stromal *pH* of illuminated chloroplasts is approximately 8.0 [17]. These data indicate that the chloroplast stroma resides in an alkaline *pH* environment and suggests that chloroplasts may encode a higher percentage of basic *pI* proteins to maintain homeostasis. The *pH* gradient between the thylakoid lumen and stroma under illuminated conditions has been reported to drive ATP synthesis, and stromal *pH* is partially dependent on the external *pH* and proton uptake by thylakoids under illuminated conditions [17, 18]. Light-induced stromal alkalization is quickly reversed under dark conditions as protons diffuse across the membrane from the thylakoid lumen. The light-induced alkaline *pH* of the stroma is crucial for the activity of photosynthetic enzymes in the carbon reduction cycle and facilitates optimal photosynthesis [19, 20]. Therefore, it is important to understand how an alkaline *pH* is maintained in the stroma of the chloroplast, which is surrounded by the acidic *pH* of the cytosol. It can be hypothesized that a complex regulatory system may exist, which is comprised of cationic/monovalent anti-porters, cation channels, and efflux carriers that transport H^+^ across the chloroplast envelope, which still remain to be identified. Chloroplasts also have the potential to generate a stromal Ca^2+^ signal in response to diverse stimuli and contribute to the fine-tuning and maintenance of stromal *pH* [21–25].

The highest percentage of basic *pI* proteins was found in protists, and the lowest percentage was found in gymnosperms. The species *Prototheca,* which lacks a chlorophyll molecule, encodes 96.428% basic *pI* proteins, while the chloroplast proteome of the parasitic plant, *Asarum minus,* possesses the highest percentage (71.428%) of acidic *pI* proteins. Due to the higher percentage of basic *pI* proteins in the chloroplast proteome, the bimodal distribution of *pI* on the proteome map falls towards the basic *pI* range (Fig. 6). Although the chloroplast proteome indicates a bimodal distribution of chloroplast proteins, the nuclear-encoded proteome in the plant kingdom exhibits a trimodal distribution [2]. Schwartz et al. (2001) reported a trimodal distribution of *pI* for eukaryotic proteins [26]. Kiraga et al. (2007) reported a bimodal distribution of the *pI* of proteins from all organisms. They indicated that taxonomy, ecological niche, proteome size, and sub-cellular localization are correlated with the presence of acidic and basic *pI* proteins [27]. Although these attributes do not show any correlation for nuclear-encoded proteins [2], the bimodal distribution of the *pI* of proteins in the chloroplast proteome is strongly correlated with the taxonomy and ecological niche of an organism (Figs. 4 and 5). The chloroplast proteome of protists and algae has a higher percentage of basic *pI* proteins, and gymnosperms have a lower percentage of basic *pI* proteins. Notably, the marine seaweed, *Prototheca stagnorum,* encodes 96.428% of its chloroplast-encoded proteins as basic *pI* proteins, reflecting the association of an ecological niche with a higher percentage of basic *pI* proteins (Supplementary file 4). In contrast, gymnosperm species were found to only encode 48.680% of its chloroplast-encoded proteins as basic *pI* proteins, reflecting the association of taxonomic rank with a higher percentage of acidic *pI* proteins.

The present study revealed that Leu was the most abundant (10.59%) amino acid in the chloroplast proteome, while Cys (1.125%) was the lowest. The chloroplast proteome’s highest and lowest abundance of amino acids was partially associated with taxonomic rank (Table 1). The chloroplast proteome of protists contained only 0.955% Cys amino acids, and algae had only 0.988%, indicating a lower abundance of Cys amino acids in lower eukaryotic plants. Leu, a non-polar amino acid, is present in chloroplast- and nuclear-encoded proteins, favoring the synthesis of non-polar amino acids rather than polar amino acids. *Pilostyles aethiopica* only contains three proteins [28] in its chloroplast proteome, which do not include any Trp amino acids (Supplementary file). The amino acid selenocysteine (Sec), which has been reported to be present in the nuclear proteome of algae and absent in all other higher plants, was not found in any of the chloroplast proteomes [2]. The selenium-containing Sec amino acid is frequently found in the proteome of animals and bacteria [29–32], where it is usually present in the active sites of protein molecules that are involved in redox reactions [31]. *Pilostyles aethiopica,* a myco-heterotrophic fungus, and an ectoparasitic land plant, has almost lost its proteome entirely. The endoparasitic flowering plant, *Rafflesia lagascae*, appears to lack a plastome [28]. The abundance of an aromatic ring containing amino acids, Trp and Tyr, is relatively low in both nuclear and chloroplast proteomes, and the complete absence of Trp in the chloroplast proteome suggests that this amino acid has undergone stringent selection pressure.

## Conclusion

Analysis of the chloroplast proteome of 2893 species of the plant kingdom revealed a diverse range of molecular mass and *pI* in chloroplast proteins. Basic *pI* proteins were dominant over acidic *pI* proteins in the chloroplast proteome, while only 0.054% neutral *pI* proteins were identified, suggesting that proteins with a neutral *pI* are rarely needed. The *pI* of chloroplast proteins covers almost the entire *pH* range (2.854–12.954). Understanding the function of these high and low *pI* chloroplast proteins will be interesting. The relative abundance of acidic and basic *pI* proteins in a chloroplast proteome is related to an organism’s taxonomic rank and ecological niche. The high and low abundance of different amino acids in the chloroplast proteome of other species may be helpful to understanding the functional role of high and low abundant amino acids in the proteome. The rate of mutation and selection pressure may be the main reasons underlying amino acid composition in the chloroplast proteome of different plant species. The presence of ambiguous amino acids Xaa, B, and J in the chloroplast proteome is intriguing and requires further investigation to understand their functional significance. In addition, the absence of Trp in the chloroplast proteome of the mycoparasitic plant, *Pilostyles aethiopica,* is also quite exciting and warrants further investigation.

## Materials and Methods

### Sequence Retrieval and Determination of Molecular Weight Isoelectric Points of Chloroplast Proteins

All the protein sequences of the chloroplast proteomes were downloaded from the National Center for Biotechnology Information (NCBI). After collecting all the protein sequences, the isoelectric point and molecular weight of the proteins were calculated using the Linux-based program of isoelectric point calculator (http://isoelectric.org/) [33]. This resulted in isoelectric point, and molecular weight files of proteins of individual species were further proceeded to remove the amino acid sequences and collected the molecular weight and isoelectric point values. The clear file of molecular weight and isoelectric point of individual species were analyzed for the amino acid count and sequence length of individual protein sequences using Linux-based command lines.

### Statistical Analysis of the Chloroplast Proteomes

All the isoelectric point and molecular weight files of the individual species were subjected to further statistical analysis. The average of protein sequences per proteome, *pI*, mol. Weight, amino acid composition, number of amino acids per sequence, and others were calculated using Microsoft excel 2016. The probability distribution of molecular weight and the isoelectric point was analyzed using an online statistical tool math portal (https://www.mathportal.org/). The scatter plot graph of the molecular weight vs isoelectric point of the chloroplast proteins was drawn using the scatterplot online server (https://scatterplot.online/). The principal component analysis of the chloroplast proteomes was conducted using the statistical tool unscrambler v 3 (https://www.camo.com/unscrambler/). Pearson’s correlation regression (*p* < 0.05) of the chloroplast proteins was analyzed using the statistical tool JASP 0.14.0.0.

## Supplementary Information

**Additional file 1: Supplementary Table 1.** Summary Statistics of molecular mass and isoelectric point (*pI*) of chloroplast proteomes.

**Additional file 2: Supplementary file 1.** Molecular mass and isoelectric point (*pI*) of chloroplast proteins.

**Additional file 3: Supplementary file 2.** Amino acid counts per proteome in chloroplast proteomes.

**Additional file 4: Supplementary file 3.** Lineage-specific molecular mass (kDa) of chloroplast proteomes.

**Additional file 5: Supplementary file 4.** Percent abundance of acidic and basic *pI* proteins in chloroplast proteomes.

**Additional file 6: Supplementary file 5.** Statistical parameters of chloroplast proteomes. The file contains the classification, number of sequences per chloroplast proteome, highest and lowest molecular mass protein in each proteome, and highest and lowest *pI* protein in the chloroplast proteome of each species. Also included are the corresponding names, numbers, percentage, and names of the highest and lowest *pI* proteins.

**Additional file 7: Supplementary file 6.** Species-wise amino acid composition of chloroplast proteomes.

## Data Availability

All the data associated with this study was taken from the publicly available database National Center for Biotechnology Information (https://www.ncbi.nlm.nih.gov/), and the accession number of the data associated with the manuscript is provided in Supplementary file 1.

## Abbreviations

*pI*: Isoelectric point
kDa: Kilo Dalton,
2D: Two-dimensional
Ala: Alanine
Cys: Cysteine
Asp: Aspartic acid
Glu: Glutamic acid
Phe: Phenylalanine
Gly: Glycine
His: Histidine
Ile: Isoleucine
Lys: Lysine
Leu: Leucine
Met: Methionine
Asn: Asparagine
Pro: Proline
Gln: Glutamine
Arg: Arginine
Ser: Serine
Thr: Threonine
Trp: Tryptophan
Tyr: Tyrosine
Val: Valine
Sec: Selenocysteine
Pyl: Pyrrolysine
CDS: Coding DNA sequence
IEF: Isoelectric focusing
IPG: Immobilized *pH* gradient
PAGE: Polyacrylamide gel electrophoresis

## References

[1] Mohanta TK, Mishra AK, Khan A, Hashem A, Abd Allah EF, Al-Harrasi A (2020). Gene loss and evolution of the Plastome. Genes (Basel).

[2] Mohanta TK, Khan AL, Hashem A, Abd Allah EF, Al-Harrasi A (2019). The molecular mass and isoelectric point of plant proteomes. BMC Genomics.

[3] Butterfield N (2000). Bangiomorpha pubescens n. gen., n. sp.: implications for the evolution of sex, multicellularity, and the Mesoproterozoic/Neoproterozoic radiation of eukaryotes. Paleobiology..

[4] Mohanta TK, Pudake RN, Bae H. Genome-wide identification of major protein families of cyanobacteria and genomic insight into the circadian rhythm. Eur J Phycol. 2017;52(2):149–65.

[5] Leister D (2003). Chloroplast research in the genomic age. Trends Genet.

[6] Farrell MJ, Reaume RJ, Pradhan AK (2017). Visual detection of denatured glutathione peptides: A facile method to visibly detect heat stressed biomolecules. Sci Rep.

[7] Su M, Ling Y, Yu J, Wu J, Xiao J (2013). Small proteins: untapped area of potential biological importance. Front Genet.

[8] Setlow P (2007). I will survive: DNA protection in bacterial spores. Trends Microbiol.

[9] Schalk C, Cognat V, Graindorge S, Vincent T, Voinnet O, Molinier J (2017). Small RNA-mediated repair of UV-induced DNA lesions by the DNA DAMAGE-BINDING PROTEIN 2 and ARGONAUTE 1. Proc Natl Acad Sci U S A.

[10] Xue Y, Shen L, Cui Y, Zhang H, Chen Q, Cui A (2013). Tff3, as a novel peptide, regulates hepatic glucose metabolism. PLoS one. Public library of. Science.

[11] Ludovic W, Jérôme B, Jian-Miao L, Delphine M, Ebrahimian GT, José V (2006). Tetrapeptide AcSDKP induces Postischemic neovascularization through monocyte chemoattractant protein-1 signaling. Arterioscler Thromb Vasc Biol.

[12] Goldstein JL, Brown MS, Stradley SJ, Reiss Y, Gierasch LM (1991). Nonfarnesylated tetrapeptide inhibitors of protein farnesyltransferase. J Biol Chem.

[13] Farwick M, Grether-Beck S, Marini A, Maczkiewitz U, Lange J, Köhler T (2011). Bioactive tetrapeptide GEKG boosts extracellular matrix formation: in vitro and in vivo molecular and clinical proof. Exp Dermatol.

[14] Lazazzera BA, Solomon JM, Grossman AD (1997). An exported peptide functions Intracellularly to contribute to cell density signaling in B. subtilis. Cell.

[15] Ramírez-Sánchez O, Pérez-Rodríguez P, Delaye L, Tiessen A (2016). Plant proteins are smaller because they are encoded by fewer exons than animal proteins. Genomics Proteomics Bioinformatics.

[16] Huber SC (1979). Effect of pH on chloroplast photosynthesis. Inhibition of O2 evolution by inorganic phosphate and magnesium. Biochim Biophys Acta.

[17] Song C-P, Guo Y, Qiu Q, Lambert G, Galbraith DW, Jagendorf A (2004). A probable Na+(K+)/H+ exchanger on the chloroplast envelope functions in pH homeostasis and chloroplast development in Arabidopsis thaliana. Proc Natl Acad Sci U S A.

[18] Mitchell P (1961). Coupling of phosphorylation to electron and hydrogen transfer by a chemi-osmotic type of mechanism. Nature.

[19] Werdan K, Heldt HW, Milovancev M (1975). The role of pH in the regulation of carbon fixation in the chloroplast stroma. Studies on CO2 fixation in the light and dark. Biochim Biophys Acta.

[20] Heldt HW, Werdan K, Milovancev M, Geller G (1973). Alkalization of the chloroplast stroma caused by light-dependent proton flux into the thylakoid space. Biochim Biophys Acta.

[21] Mohanta KT, Yadav D, Khan LA, Hashem A, Abd Allah FE, Al-Harrasi A (2019). Molecular players of EF-hand containing calcium signaling event in plants. Int J Mol Sci.

[22] Mohanta TK, Bashir T, Hashem A, Abd Allah EF, Khan AL, Al-Harrasi AS (2018). Early events in plant abiotic stress signaling: interplay between calcium, reactive oxygen species and Phytohormones. J Plant Growth Regul.

[23] Mohanta TK, Mohanta N, Mohanta YK, Bae H (2015). Genome-wide identification of calcium dependent protein kinase gene family in plant lineage shows presence of novel D-x-D and D-E-L motifs in EF-hand domain. Front Plant Sci.

[24] Mohanta T, Kumar P, Bae H (2017). Genomics and evolutionary aspect of calcium signaling event in calmodulin and calmodulin-like proteins in plants. BMC Plant Biol.

[25] Navazio L, Formentin E, Cendron L, Szabò I (2020). Chloroplast calcium signaling in the spotlight. Front Plant Sci.

[26] Schwartz R, Ting CS, King J (2001). Whole proteome pl values correlate with subcellular localizations of proteins for organisms within the three domains of life. Genome Res.

[27] Kiraga J, Mackiewicz P, Mackiewicz D, Kowalczuk M, Biecek P, Polak N (2007). The relationships between the isoelectric point and: length of proteins, taxonomy and ecology of organisms. BMC Genomics.

[28] Bellot S, Renner SS (2015). The plastomes of two species in the endoparasite genus pilostyles (Apodanthaceae) each retain just five or six possibly functional genes. Genome Biol Evol.

[29] Böck A, Forchhammer K, Heider J, Leinfelder W, Sawers G, Veprek B (1991). Selenocysteine: the 21st amino acid. Mol Microbiol.

[30] Mousa R, Notis Dardashti R, Metanis N (2017). Selenium and selenocysteine in protein chemistry. Angew Chemie Int Ed.

[31] Zhang Y, Romero H, Salinas G, Gladyshev VN (2006). Dynamic evolution of selenocysteine utilization in bacteria: a balance between selenoprotein loss and evolution of selenocysteine from redox active cysteine residues. Genome Biol.

[32] Novoselov SV, Rao M, Onoshko NV, Zhi H, Kryukov GV, Xiang Y (2002). Selenoproteins and selenocysteine insertion system in the model plant cell system, Chlamydomonas reinhardtii. EMBO J.

[33] Kozlowski LP (2016). IPC – Isoelectric Point Calculator. Biol Direct.

