# Supplementary material for: Decoding the Virtual 2D Map of the Chloroplast Proteomes
Source: Biol Proced Online. 2022 Dec 13;24:23. doi: 10.1186/s12575-022-00186-8 (PMC9749319; doi:10.1186/s12575-022-00186-8)
Supplement: Supplementary file 1 — Additional file 1: Supplementary Table 1. Summary Statistics of molecular mass and isoelectric point (pI) of chloroplast proteomes. [file 12575_2022_186_MOESM1_ESM.docx]

Supplementary Table 1

Summary Statistics of molecular weight and isoelectric point (pI) of chloroplast proteomes.

| **Statistical Parameters** | **Isoelectric Point (*pI*)** | **Molecular Weight** |
| --- | --- | --- |
|  |  |  |
| N | 256387 | 256387 |
| Min | 2.854 | 0.448 |
| Max | 12.954 | 616.334 |
| Mean | 7.852 | 32.483 |
| Std. error | 0.0046 | 0.087 |
| Variance | 5.613 | 1966.947 |
| Stand dev. | 2.369 | 44.35 |
| Median | 7.951 | 17.669 |
| 25 prcntl | 5.715 | 9.18 |
| 75 prcntil | 9.736 | 38.95 |
| Skewness | 0.108 | 3.569 |
| Kurtosis | -1.246 | 15.282 |
| Geom. Mean | 7.481 | 18.727 |
| Coeff. Var | 30.172 | 136.533 |

| **Normal Distributions** | |
| --- | --- |
| *pI* | |
| *P* (*X >*12.954) | 0.0158 |
| *P* (*X <* 2.854) | 0.0174 |
| *P* (*X >* 7.951) | 0.484 |
| *P* (*X <* 7.951) | 0.516 |
| Mol. Weight | |
| *P* (*X >* 616.334) | 0 |
| *P* (*X <* 0.448) | 0.235 |
| *P* (*X >* 17.669) | 0.629 |
| *P* (*X <* 17.669) | 0.370 |
|  |  |
